# Neonatal Outcome After Expectant Management of Preterm Premature Rupture of Membranes (PPROM) Between 34+0 and 36+6 Weeks of Gestation: A Single-Center Cohort Study

**DOI:** 10.7759/cureus.92985

**Published:** 2025-09-22

**Authors:** Amrei Welp, Sara Christesen, Jann Lennard Scharf, Christoph Dracopoulos, Perke Pricker, Achim Rody, Jan Weichert, Michael Gembicki

**Affiliations:** 1 Department of Gynecology and Obstetrics, Division of Prenatal Medicine, University Hospital of Schleswig-Holstein, Campus Luebeck, Luebeck, DEU; 2 Department of Gynecology and Obstetrics, University Hospital of Schleswig-Holstein, Campus Luebeck, Luebeck, DEU; 3 Prenatal Medicine, Elbe Center of Prenetal Medicine and Human Genetics, Hamburg, DEU

**Keywords:** expectant management, late preterm pregnancies, peripartum complications, preterm premature rupture of membranes, prevent chorioamnionitis

## Abstract

Objectives: The aim of the study was to assess adverse neonatal outcome after preterm premature rupture of membranes (PPROM) between 34+0 and 36+6 weeks of gestation in patients undergoing expectant management after PPROM according to the intrauterine inflammation, infection, or both (TRIPLE-I) criteria.

Study design: This retrospective analysis included 323 singleton pregnancies with PPROM between 34+0 and 36+6 weeks of gestation. Groups of cases that met at least some of the TRIPLE-I diagnostic criteria and were suspected of having TRIPLE-I were created and compared with groups of cases that did not meet these criteria.

Results: Mean gestational age at time of birth was 35.9 weeks [IQR; 35.0-36.4], mean gestational age at time of PPROM was 34.7 [IQR; 34.0 -35.4], and mean birth weight was 2,660 g [IQR; 2,140-2,985]. Two hundred four (63.2%) infants were delivered vaginally, 107 (33.1%) via caesarean section and 12 (3.7%) women had vaginal operative delivery (vacuum extraction). There were only two cases of maternal fever, and no case met all TRIPLE-I diagnostic criteria. No significant increase in risks were found for low umbilical artery pH (OR: 1.88 (95% CI: 0.50-5.88), p = 0.29), low Apgar score (OR: 1.14 (95% CI: 0.17-4.73), p = 0.86), or need for neonatal admission to neonatal intensive care unit (NICU) due to infection (OR: 1.14 (95% CI: 0.17-4.73), p = 0.62) in women with elevated white blood cell count in performed logistic regressions. Same applied for cases with tachycardic cardiotocography (CTG).

Conclusion: Expectant management appears to be a viable and potentially safe approach for managing cases of PPROM between 34+0 and 36+6 weeks of gestation, without significantly increasing the risk of adverse neonatal outcome. However, close monitoring, personalized care and readiness to intervene are important for optimally managing these clinical cases.

## Introduction

Preterm premature rupture of membranes (PPROM) occurs after 34 weeks' gestation with an incidence of about 1.5% [1]. At this relatively late stage of pregnancy, treatment with betamethasone to mature the lungs of the fetus and tocolysis to prolong pregnancy are no longer indicated [2,3].

Recommendations in international guidelines concerning the optimal pregnancy management in those cases are inconsistent. Some guidelines equate immediate induction of labor (to prevent infection and the potential risk of stillbirth) with expectant management [4-8] and suggest that both options should be discussed with the parents. Others recommend expectant management up to 37 weeks' gestation [7-9] since induction of labor may lead to preterm birth and increase neonatal complications [1] (see Appendix 1). There is consensus that prophylactic antibiotic treatment should be given in all cases [4-9]. Based on the PPROMT, PPROMEXIL and PPROMEXIL-2 trial [10,11], in which the risk of neonatal sepsis after late PPROM (after 34 weeks) was found to be low and induction of labor did not reduce it [10], expectant management is the preferred approach in many cases [1,7-9,12]. In addition, with regard to the role of microbiome, intraamniotic inflammation associated with microbial ascendancy has recently been shown to decrease with advantageous stages (>34 weeks) of pregnancy in patients with preterm labor [13]. Nevertheless, clinical signs of chorioamnionitis or intrauterine inflammation, infection, or both (TRIPLE-I) such as maternal fever or tachycardic cardiotocography (CTG) after PPROM, should lead to a prompt delivery, as chorioamnionitis increases the known risks of adverse outcome (e.g. neonatal sepsis, endomyometritis, wound infection) [14].

The aim of this retrospective analysis was to assess whether expectant management after late PPROM (34+0 to 36+6 weeks) was associated with neonatal infections and whether the diagnostic criteria of TRIPLE-I (maternal fever, maternal elevated white blood cell count and tachycardic CTG) were associated with an increased risk of adverse neonatal outcome (low Apgar score, low umbilical artery pH and admission to neonatal intensive care unit (NICU) due to suspected infection).

## Materials and methods

Study population

This study included 323 women at a single tertiary center (University Hospital of Schleswig-Holstein, Campus Luebeck, Germany) who delivered their babies between January 2019 and October 2024 after PPROM between 34+0 and 36+6 weeks of gestation. To analyze only singleton late preterm infants, women with PPROM and delivery before 34+0 weeks were excluded, as were deliveries close to term (after 36+6 weeks) and twin pregnancies. All patients received 2 g ampicillin intravenously three times a day (every eight hours) for seven days as soon as possible after PPROM.

Data on previous medical history, mode of delivery, indication for delivery, changes in CTG and maternal white blood cell count were recorded for the pregnant women based on electronic patient records in ViewpointTM 6, Trium CTG online and Agfa Orbis. Neonatal birth weight, Apgar score, umbilical artery pH, NICU admission and need for antibiotic treatment were recorded for the newborns.

Definitions

We defined PPROM as leakage of amniotic fluid proven by biochemical test (e.g. Amnisure). Adverse neonatal outcome was defined as umbilical artery pH less than 7.20, Apgar score less than 7 at five minutes and NICU admission for suspected infection. Potential risk factors were defined as a maternal white blood cell (WBC) count greater than 15,000/µl, a tachycardic CTG persisting more than 30 minutes and maternal fever subpartum according to TRIPLE-I diagnostic criteria [15]. Maternal fever was defined as body temperature above 38.0°C persisting over 30 minutes [7]. There were only two cases of maternal fever, which we therefore decided not to consider as a risk factor in this analysis, due to the low number of cases. The Apgar score was measured according to standard criteria based on Virginia Apgar [16]. Due to its prognostic relevance, we used the five-minute value for analysis. We excluded antibiotic treatment in the NICU, because antibiotics were administered generously in our NICU without strong suspicion of infection. Four cases were excluded; three delivered their baby at another hospital and one incomplete data set was found and excluded.

Ethics

The study was approved by the local ethics committee (Ethikkommission der Universitaet zu Luebeck, Germany) in July 2025, number 2025-347 and a signed broad consent form was obtained from all patients.

Statistical analysis

GraphPad Prism 10 for Mac (version 10.4.1, GraphPad Software Inc., La Jolla, CA, USA) and Microsoft Excel for Mac (version 16.69.1, Microsoft Corp., Redmond, WA, USA) were used. Descriptive statistics, chi-squared tests, and logistic regressions were used to show associations and increased risks between the above risk factors and outcomes. The type I error level was set at 0.05. P values < 0.05 were considered significant. Incomplete records were excluded before analysis.

## Results

Clinical characteristics

A total of 323 cases were included in the final analysis, after the exclusion of women who gave birth after 36 weeks and six days of gestation, and of women with twin pregnancies. The mean maternal age was 32.0 years (IQR: 28.0-36.0), the mean gestational age (GA) at delivery was 35.9 weeks (IQR: 35.0-36.4), mean gestational age at PPROM was 34.7 weeks (IQR: 34.0-35.4) and the mean birth weight was 2,660 g (IQR: 2,140-2,985). The mean Apgar score at five minutes was 9 (IQR: 8-10), and the mean umbilical cord pH was 7.26 (IQR: 7.19-7.32) (see Table 1). All the women received expectant management, and no induction of labor was performed. Most women delivered vaginally (n = 204, 63.2%), while about one third had a caesarean section (n = 107, 33.1%). The most common indications were fetal breech presentation (n = 31, 33.2%) and previous caesarian section (n = 25, 26.7%) (see Table 2). Only a few women have had a caesarean section because of suspected infection (n = 11, 11.8%). Twelve women (3.7%) had an assisted vaginal birth with vacuum extraction (see Table 2). We only found two women with a fever during labor, one delivered via caesarean section and the other had a vaginally assisted birth.

**Table 1:** Population characteristics WBC – white blood cell count; BE – base excess; NICU – neonatal intensive care unit; TIPLE-I – intrauterine infection, inflammation or both

Clinical characteristics	median [IQR]
Maternal Age (years)	32.0 [29 – 36]
Gestational age at birth (weeks)	35.9 [35 – 36.4]
Birth weight (gram)	2,660 [2,410 – 2,985]
Maternal WBC (10^3^/µl)	11.65 [9.67 – 13.91]
APGAR 5 min (0-10)	9 [8 – 10]
Umbilical artery pH (7.00 – 7.40)	7.26 [7.19 – 7.32]
Umbilical artery BE (0 – (-15))	- 4.6 [- 6.9 – (-2.05)]

**Table 2:** Mode of delivery and indications for caesarian section NICU – neonatal intensive care unit; TIPLE-I – intrauterine infection, inflammation or both

Delivery mode and indications for caesarian section	%/n
Spontaneous vaginal delivery	63.2 / 204
Vaginal assisted delivery	3.7 / 12
Admission to NICU due to suspected infection	11 / 26
Caesarian section	33.1 / 107
Breech position	33.2 / 31
Transverse position	2.1 / 2
Birth arrest	12.8 / 12
Pathologic cardiotocography	13.9 / 13
Suspected TRIPLE-I	11.8 / 11
Placental abruption	2.1 / 2
Fetal growth restriction	3.2 / 3
Previous caesarian section	26.8 / 25
Fibroids	1.1 / 1
Maternal request	7.5 / 7

Comparison of groups according to TRIPLE-I diagnostic criteria

**Figure 1:**
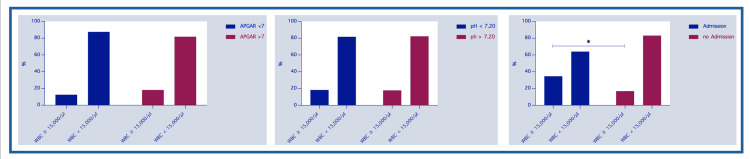
Percental number of infants stratified to Apgar score at five minutes, umbilical cord pH and admission to NICU born to mothers with elevated or normal WBC; * statistically significant NICU – neonatal intensive care unit; WBC – white blood cell count

The chi-squared test results revealed several findings regarding maternal WBC count, fetal heart rate pattern, and need for neonatal admission. In the group of infants with an umbilical artery pH below 7.20 (n = 83), 18.3% of mothers (15 out of 83) had elevated WBC counts, while the majority (81.7%; 67 out of 83) had normal WBC counts (see Figure 1). Only a small number of these cases (2.1%; 2 out of 83) showed a tachycardic CTG. Among infants with an Apgar score of less than 7 at five minutes (n = 9), just one mother (11.1%) had an elevated WBC count, while eight mothers (88.9%) had normal WBC counts. In this group, a tachycardic CTG was observed in one case (11.1 %).

**Figure 2:**
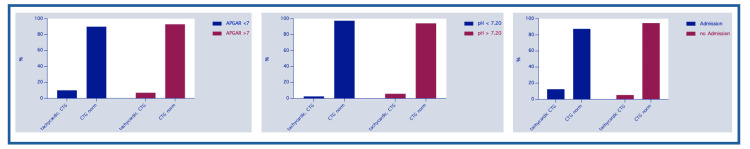
Percental number of infants stratified to Apgar score at five minutes, umbilical cord pH and admission to NICU, born to mothers with tachycardic or normal CTG. CTG – cardiotocography, NICU – neonatal intensive care unit

Among the 26 newborns who were admitted to the NICU due to suspected infection, 34.6% (9 out of 26) were born to mothers with elevated WBC counts, whereas 65.4% (17 out of 26) were born to mothers with normal WBC counts. Additionally, 12.5% (3 out of 26) of these mothers had a tachycardic CTG during labor (see Figure 2), while the remaining 87.5% (23 out of 26) had normal CTGs (see Table 3).

**Table 3:** Distribution of cases according to adverse outcomes Legend:  df – degrees of freedom; WBC – white blood cell count; CTG – cardiotocography; UC – umbilical cord *statistically significant Reference ranges: pH (7.00 – 7.40), APGAR 5min (0-10), WBC < 15,000/ul Values are based on chi-squared tests calculated with GraphPad Prism 10 for Mac (version 10.4.1, GraphPad Software Inc., La Jolla, CA, USA), type I error level was set at 0.05. P values < 0.05 were considered significant.

	WBC ≥ 15,000/ul	WBC < 15,000/ul	tachycardic CTG	normal CTG	chi-square value	df	P-value
			% / n	%/n			
UC pH < 7.20	18.3 / 15	81.7 / 67			0.008	1	0.92
(n = 83)			2.1 / 2	97.9 / 81	2.246	1	0.13
Apgar score < 7 (n = 9)	11.1 / 1	88.9 / 8			0.2945	1	0.58
			11.1 / 1	90 / 9	0.5582	1	0.45
Admission to NICU	34.6 / 9	65.4 /17			5.326	1	0.02*
(n = 26)			12.5 / 3	87.5 / 23	2.051	1	0.15

A significant difference between the groups with and without elevated WBC counts was only found among infants admitted to the NICU due to suspected infection (p = 0.02). No significant differences were found between any of the other groups (see Table 3).

Logistic regression and odds ratios

Logistic regression showed an odds ratio (OR) of 1.33 (95% confidence interval (CI): 0.37-3.87) and a p-value of 0.62 for NICU admission in the group with elevated WBC count, and an OR of 2.83 (95% CI: 0.98-10.22) and a p-value of 0.07 in the group with tachycardic CTG. For an umbilical artery pH of less than 7.20, the OR was 1.88 (95% CI: 0.50-5.88) and the p-value was 0.29 in the group with elevated WBCs, compared to an OR of 0.37 (95% CI: 0.06-1.36) and a p-value of 0.19 in the group with tachycardic CTG. For an Apgar score of less than 7 points at five minutes, the OR was 1.14 (95% CI: 0.17 - 4.73) in the group with elevated WBC count, and the OR was 1.76 (95% CI; 0.83 - 1.41) with a p-value of 0.62 in the group with tachycardic CTG (see Table 4). No significant increase in the risk of any adverse outcome (admission to NICU, pH <7.20 or Apgar score of less than 7 at five minutes) was found in either group. Again, we did not perform an analysis with maternal fever because only two cases were found in the analyzed study group.

**Table 4:** Odds ratios of risk factors Legend: UC – umbilical cord; WBC – white blood cell count; CTG – cardiotocography; (log-odds) – regression coefficient in log-odds unit; NICU – neonatal intensive care unit OR- odds ratio; SE – standard error Reference ranges: pH (7.00 – 7.40), APGAR 5min (0-10), WBC < 15,000/ul Values are based on simple logistic regressions calculated with GraphPad Prism 10 for Mac (version 10.4.1, GraphPad Software Inc., La Jolla, CA, USA), type I error level was set at 0.05. P values < 0.05 were considered significant.

Outcome	Risk factor	ß (log-odds)	SE	OR [95% CI];	P value
UC pH < 7.20					
	WBC ≥ 15,000/ul	0.636	0.610	OR 1.88 [0.50 – 5.88]	0.29
	tachycardic CTG	-0.981	0.763	OR 0.37 [0.06 – 1.36]	0.19
	Apgar score < 7					
WBC ≥ 15,000/ul		0.137	0.804	OR 1.14 [0.17 – 4.73]	0.86
tachycardic CTG		0.563	1.075	OR 1.76 [0.09 – 19.92]	0.62
Admission to NICU						
	WBC ≥ 15,000/ul	0.283	0.586	OR 1.33 [0.37 – 3.87]	0.62
	tachycardic CTG	1.041	0.583	OR 2.83 [0.98 – 10.22]	0.07

## Discussion

This retrospective, single-center study examined the neonatal outcome of expectant management following PPROM between 34+0 and 36+6 weeks of gestation. Our findings contribute to current discussions concerning the optimal clinical management of these patients, balancing the risks of infection, clinical chorioamnionitis and prematurity [13].

Several recent studies and guidelines consider expectant management between 34 and 37 weeks of gestation to be a safe alternative to immediate delivery or induction of labor if there are no signs of infection or other maternal or fetal indications [1,17,18]. Notably, we found no cases of suspected infection that met the TRIPLE-I criteria [15]. Only two of the 323 women in this study had fever. There was no significant increase in suspected neonatal infection or sepsis, and the number of neonatal admissions for this reason was low (11%). A substantial proportion of women achieved additional gestational maturity (the mean GA at birth was 35.9 weeks, mean GA at PPROM 34.7), which is associated with improved neonatal outcome [17,19,20]. In this gestational window, previous randomized controlled trials (e.g. the PPROMEXIL and PPROMEXIL-2 trials [10,11]) and meta-analyses have shown similar results when comparing active management with expectant management. Most of these studies did not report an increase in adverse neonatal outcome with expectant management [10,11,21]. However, Bond et al. [17] reported an increased risk of caesarean section and respiratory distress syndrome in cases where labor was induced. However, we could not replicate these findings. The caesarean section rate in this study group was like the overall rate during the same period, with only a small minority undergoing a caesarean section due to suspected chorioamnionitis. Despite the partial presence of TRIPLE-I diagnostic criteria in some cases, our results showed no significant difference in adverse perinatal outcome (low umbilical cord pH, low Apgar score, admission to NICU). Nevertheless, the decision to proceed with expectant management after PPROM between 34 and 37 weeks of gestation should consider gestational age, signs of infection, fetal weight, blood circulation, doppler ultrasound, and the availability of neonatal intensive care [21]. The data from this retrospective analysis support the recommendations of the German guideline (AWMF) and NICE guidelines, which endorse expectant management after PPROM up to 37 weeks of gestation only, if signs of clinical chorioamnionitis are absent [22]. In this context diagnosing chorioamnionitis after PPROM remains challenging, as the clinical presentation is often subtle or non-specific. Preterm pregnancies often lack the classic signs of infection, such as maternal fever, uterine tenderness or foul-smelling amniotic fluid [23]. If present, maternal fever [24,25], leukocytosis and inflammatory markers are rather non-specific and may overlap with other causes of inflammation [26,27]. In our study cohort the admission rate was lower in this subgroup, which can be explained by the small group size and the low specific value of white blood cell count in pregnancy. This emphasizes the need for a definite diagnosis of chorioamnionitis which is made by analyzing amniotic fluid to detect bacteria and inflammatory proteins [22] or postnatally based on histopathological findings. For this analysis, we used the diagnostic criteria for TRIPLE-I, as listed in the German guideline [7]. Based on our data, as well as on similar findings from literature [26,28,29], it can be debated whether a more complex case definition, as presented by Kachikis et al. [30], would allow for a more accurate diagnosis and subsequent management. In clinical practice, careful monitoring, appropriate antibiotic treatment, and timely intervention remain essential to minimize the risk of infectious complications for both mother and fetus [18].

Strengths and limitations

Our study population reflecting the actual clinical practice in a large single tertiary center is a clear strength of the study. However, this design also brings some limitations, including the retrospective design, a potential selection bias and the incapability to control for all confounding factors. Due to the small number of adverse outcomes, there is limited statistical power, which results in wide confidence intervals and increases the risk of type II error. Therefore, caution is certainly needed when interpreting the negative findings. Excluding maternal fever as a predictor from logistic regression analysis may affect the generalizability of our findings and the validity of applying the full TRIPLE-I definition. Also, the generalizability of our findings may be limited to the relatively small sample size in a single-center setting. In addition, there is no data on long-term neonatal outcome and subtle differences in morbidity may be underestimated.

## Conclusions

In conclusion, this analysis supports the feasibility and general safety of expectant management after PPROM between 34 and 37 weeks of gestation, without a statistically significant increase in adverse neonatal outcomes in our cohort. However, given the relatively small number of adverse events and limited statistical power, these findings should be interpreted with caution. Close monitoring, individualized care and the readiness to intervene remain essential components of an optimal management of these clinical cases. Further prospective multicenter studies with larger sample sizes and follow up data are needed to define a risk stratification and to identify subgroups that may benefit most from expectant management.

## Appendices

**Table 5:** Supplementary Table: Overview of Guidelines and Recommendations AWMF: Association of Scientific Medical Societies in Germany, CNGOF: French College of Gynecologists and Obstetricians, NICE: National Institute for Health and Care Excellence, ACOG: American College of Obstetricians and Gynecologists

Guideline	Recommendation
AWMF	Favors expectant management until 37+0 weeks for uncomplicated cases (absent infection)
CNGOF	Favors expectant management until 37+0 weeks for uncomplicated cases (absent infection)
NICE	Individual decision for induction of labor or expectant management after counseling the parents
ACOG	Individual decision for induction of labor or expectant management after counseling the parents
